# Biochemical Markers Involved in Bone Remodelling During Orthodontic Tooth Movement

**DOI:** 10.3390/jfb17010007

**Published:** 2025-12-22

**Authors:** Beatriz Patricia Fuentes Vera, Ibrahim Dib Zaitun, María Ángeles Pérez de la Cruz

**Affiliations:** 1 Department of Anatomy and Histology, University of Salamanca, 37003 Salamanca, Spain; mapec@usal.es; 2 Department of Surgery, University of Salamanca, 37003 Salamanca, Spain; ibrahimdib@usal.es

**Keywords:** bone remodelling, orthodontic tooth movement, biochemical markers, osteoblasts, osteoclasts, osteocytes, osteoprotegerin, RANKL, cytokines, molecular diagnostics

## Abstract

Bone remodelling is a physiological process influenced by mechanical stimuli such as those generated during orthodontic treatment. Biochemical markers allow the phases of remodelling to be identified, its progression to be assessed, alterations to be detected and scaffold-based tissue regeneration to be evaluated. This study reviews the main markers involved in bone formation and resorption, highlighting their clinical relevance. A literature search was conducted in biomedical databases, selecting studies that analysed crevicular gingival fluid samples in areas of tension and compression. The markers were classified according to their function and location, and their baseline values, temporal variations and methods of analysis were compiled. Among the markers of bone formation, Osteoprotegerin (OPG), Transforming Growth factor β1 (TGF-β1) and Interleukin 27 (IL-27) stand out; while resorption markers include Receptor Activator of Nuclear Factor appa β Ligand (RANKL), Tumour Necrosis Factor (TNF-α) and Interleukin 1β (IL-1β). The results show different expression patterns depending on the type of force applied and the timing of the follow-up, allowing molecular profiles associated with each phase of remodelling to be established. This characterisation improves our understanding of tooth movement and provides a basis for the development of more precise scaffolds and functional biomaterials in orthodontics.

## 1. Introduction

Bone remodelling is a dynamic process that is essential for maintaining the structural integrity of the skeleton. It is based on two processes: bone formation and bone resorption.

In bone formation or osteogenesis, osteoblasts (OBs) fix extracellular calcium using osteocalcin, secrete alkaline phosphatase and pyrophosphatase, and generate hydroxyapatite crystals that form osteoid, a mineralised matrix rich in collagen. Some OBs become osteocytes, and others become lining cells [1,2,3,4].

In bone resorption, OBs release cytokines that activate osteoclasts (OCs). These generate Howship lacunae and degrade bone with specific enzymes. Subsequently, they release signals that induce the maturation of new OBs, which secrete Osteoprotegerin (OPG), inhibiting osteoclastic activity and closing the cycle [2,3,5,6].

**Figure 1:**
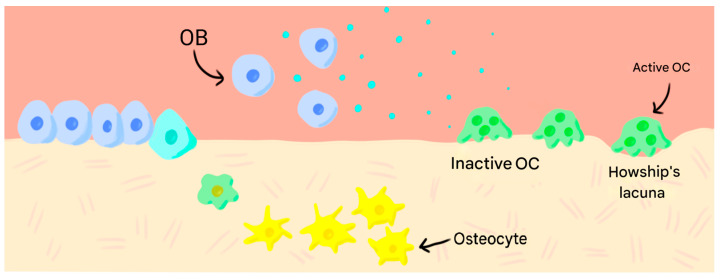
Bone remodelling process.

Through these processes, aged bone tissue is removed and subsequently replaced by new bone (Figure 1).

**Figure 2:**
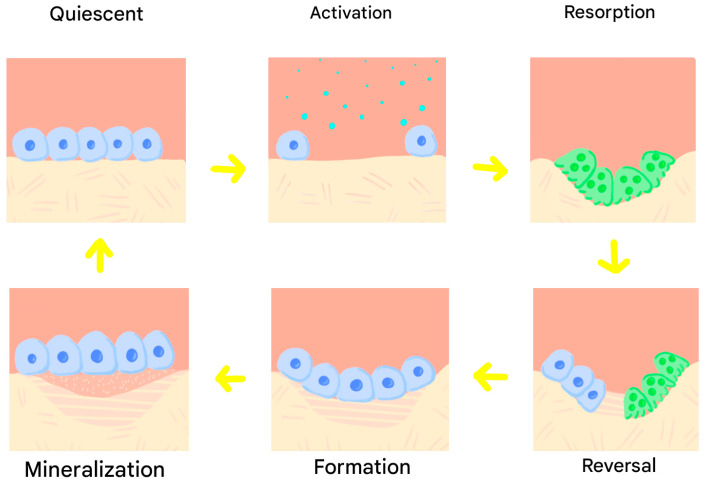
Phases of bone remodelling.

The phases of bone remodelling are divided into the quiescent phase, in which the bone remains at rest; the activation phase, in which paracrine factors are released that activate OCs and duplicate OBs precursors; the resorption phase, in which stabilised OCs degrade the bone matrix and osteoid using acids; the reversal phase, in which a cementing substance is secreted that facilitates the adhesion of OBs to the resorbed area; the formation phase, where OBs synthesise and mineralise the new osteoid matrix; the mineralisation phase, in which minerals are deposited on the osteoid; and the quiescent phase, where OBs become lining cells or remain trapped as osteocytes, restarting the cycle [7,8,9,10,11] (Figure 2).

This balance between bone destruction and formation is altered in various conditions. The factors to be taken into account are as follows: genetic, since between 60 and 80% of peak bone mass is regulated by this; mechanical, such as physical activity; vascular-nervous, Trueta, in 1963 [12], was the first to confirm that the quantity and quality of cells that can reach the bloodstream is crucial to ensuring stability in the bone remodelling process; nutritional, due to the amount of minerals obtained from the diet, especially Ca^2+^ and P; hormonal, taking into account parathyroid hormone (PTH), calcitriol and calcitonin (CT); and local, such as growth factors or cytokines [13,14,15,16,17,18,19].

Furthermore, this balance can be altered during orthodontic treatment, where mechanical forces induce cellular and immunological responses that modify the behaviour of these cells [20,21].

Tooth movement achieved through orthodontics consists of moving the teeth while seeking to minimise adverse effects on both the teeth and adjacent tissues.

In 1880, the first theory of tooth movement was established, in which Kingsley [20] stated that it was due to the elasticity of the alveolar bone. This theory is now obsolete, but it paved the way for further research, which has led to the following theories.

The pressure-tension theory, or classical theory, was established in 1932 by Sandsteds [21,22], Oppenheim [23] and Schwarz [24], who, after conducting studies on histological sections of tooth movement, concluded that thanks to the existence of pressure and tension sides in the periodontal ligament (PDL), the tooth could move through it, as this generated a reaction in which the alveolar bone could form or resorb.

The theory of piezoelectricity was described by Fukada and Yasuda [25] in 1957. This theory explains tooth movement through electrical signals that are generated when the alveolar bone flexes and deforms after the application of a certain force.

The latest theory that remains today is the hydrodynamic theory. It was proposed by Brännström [26] in 1966. This explains tooth movement through the circulation of fluid in the bone and tooth cells, where osteocytes are found, which are activated and begin to perform their functions.

**Figure 3:**
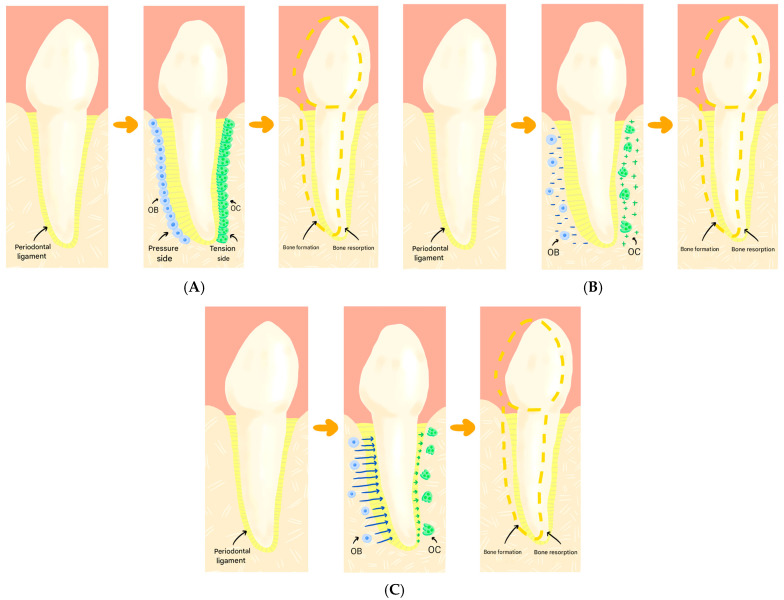
Theories of tooth movement. (**A**) Pressure-tension theory. (**B**) Piezoelectricity theory. (**C**) Hydrodynamic theory.

These theories are not mutually exclusive and can coexist, jointly explaining the phenomenon of orthodontically induced tooth movement (Figure 3).

Although the cellular regulation of bone remodelling has been extensively researched, the molecular regulation of these processes and their potential as functional biomaterials is still not entirely clear [27]. This is where biochemical markers play a fundamental role.

According to the *National Institute of Health* (NIH), biochemical markers are ‘a characteristic that is measured and evaluated objectively as an indicator of normal biological processes, pathogenic processes, or pharmacological responses to a therapeutic intervention’ [28].

Biochemical markers therefore allow us to accurately identify the phases of remodelling, evaluate its progression, detect possible alterations and use them in technologies to design scaffolds from biomaterials.

In the context of bone regeneration in orthodontics, these markers make it possible to determine whether the tissue is in the formation phase (tension side) or resorption phase (pressure side), and whether this phase is developing properly or is altered by external or pathological factors. Furthermore, this link is particularly relevant in the field of dentistry, as it could be used to evaluate biomaterials such as bioactive ceramics, polymeric scaffolds or calcium phosphate cements, which can influence the activity of OBs and OCs, and whose performance can be monitored using these biochemical markers.

Controlled tooth movement depends on three key factors: force, resistance and acceleration [29]. Although historically the emphasis has been on the application of light forces and the structural resistance of the PDL, attention has now shifted to how to achieve controlled acceleration of tooth movement in order to shorten and personalise orthodontic treatments, avoiding or reducing possible harmful effects on the oral cavity throughout this process.

This acceleration, linked to the degree of metabolic alteration, has led to the development of techniques that seek to optimise the speed of tooth movement without compromising bone biology. Consequently, the acceleration of tooth movement has become a central topic of contemporary orthodontic research.

Therefore, research into biochemical markers is essential for understanding, monitoring and optimising the bone remodelling processes involved in accelerating tooth movement.

Despite emerging experimental research to determine the biochemical markers involved in bone formation and resorption in the presence of orthodontics, there is no consensus on these markers, as many of them are not specific enough to determine their presence in these conditions.

That is why the aim of this study is to identify the main biochemical markers involved in bone remodelling during orthodontic treatment, specifically those associated with bone formation (tension side) and bone resorption (pressure side), to advance the design and evaluation of scaffold-based tissue regeneration, which represents a promising approach to optimising orthodontic treatments.

## 2. Materials and Methods

This study is based on a review of the available scientific literature on biochemical markers involved in bone remodelling during orthodontic treatment, dividing these biochemical markers into bone formation markers and bone resorption markers.

To this end, an exhaustive search was conducted in various databases such as PubMed, Embase, Cochrane Library and Scopus.

The following *Medical Subject Headings* (MeSH) search terms and consecutive Boolean operators were used in PubMed, along with the “Humans” filter: (Biomarkers OR Markers OR Mediators) AND (“Orthodontic” OR “Dental Movement”) NOT “Case Report”. The search strategy is provided in Supplementary Material S1.

There was no restriction on publication time or languages. To avoid omitting relevant articles, each database was consulted independently by two authors. Data extraction from each original article was also performed by two authors separately. The following specific variables were extracted from each study: sample size, tooth studied, type of intervention, treatment method, sample collection location, sample collection method, sample analysis method, baseline value, value of each biomarker at each time point and main outcomes. The literature search was initiated in January 2025 and concluded in September 2025. Case–control studies on biochemical markers involved in bone formation and/or resorption processes were included. Studies containing information relevant to the review were selected for full-text evaluation. Studies published as conference abstracts, editorials, opinions, preprints, theses, and literature reviews were excluded, along with those that did not collect samples in crevicular fluid (CF) and did not specify the comparative numerical data or the tooth surfaces (tension and compression) from which the samples were collected.

The reason for excluding saliva-based and blood-based studies is the nonspecificity of these media, as these samples contain a mixture of biochemical markers associated with both bone formation and resorption.

For the same reason, studies in which the sample is collected from the CF but the side from which it is collected (pressure or tension) is not specified are also excluded: the dental area presents considerable spatial limitations, where both processes occur simultaneously in a small space, making it difficult to differentiate their individual contributions if the sample is not collected directly from the CF in the area where these changes occur.

When the studies did not show comparable numerical data, but did show data in the form of graphs, these were extracted using the *WebPlotDigitizer* software in its 5th version.

**Figure 4:**
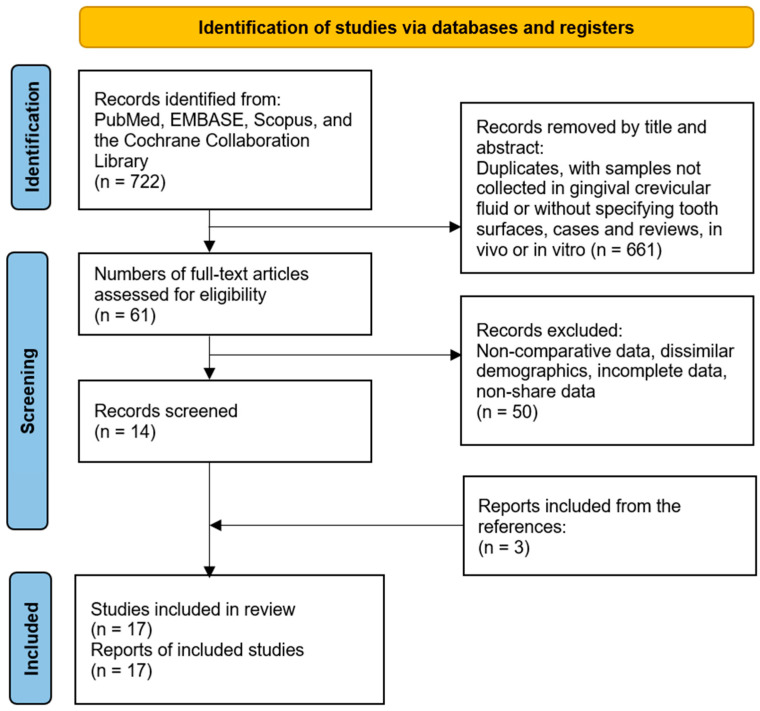
Flowchart of study selection based on the PRISMA criteria.

A flow diagram was created to visualise the selection of studies using the 2020 *Preferred Reporting Items for Systematic Reviews and Meta-Analyses* (PRISMA) guideline [30] (Figure 4).

The methodological quality of the observational studies has been independently evaluated by two reviewers using the Methodological Index for Non-Randomised Studies (MINORS) criteria [31] (Table 1). This instrument contains items to evaluate key methodological aspects such as study design, patient selection, outcome measures, and follow-up. Scoring ranges from 0 to 16 for non-comparative studies and 0–24 for comparative designs. For non-comparative studies, those scoring 0–4 will be considered very low quality, 5–7 as low quality, 8–12 as fair quality, and 13–16 as high quality. Comparative studies scoring 0–6 will be deemed very low-quality, 7–10 as low quality, 11–15 as fair quality, and 16–24 as high quality. Any discrepancies in the quality assessment scoring between the two reviewers will be discussed to reach a consensus.

## 3. Results

For a biochemical marker to be considered clinically useful, it must be specific to bone metabolism, accessible by non-invasive methods, and allow for reliable and reproducible interpretation [49,50]. This requirement is particularly important when assessing biomaterials since their effectiveness depends on measurable and reproducible biological responses.

In order to provide a structured overview of the studies included in this article, the biochemical markers studied in each of them, the sample, the follow-up carried out, the detailed procedure for obtaining and analysing, and the main results of these studies, a table has been created containing detailed information on each of them (Table 2).

In order to focus on which biochemical markers were specific to each bone process in orthodontic movements, they are divided into two large groups, depending on where they are found: bone formation markers, located on the dental tension side of the orthodontic movement; and bone resorption markers, located on the dental compression side of this movement. Within these, their definition, their main functions in bone regeneration, and the phase in which their concentration is highest will be described. These phases are as follows: early, in which the maximum concentration of the biochemical marker occurs before the first 24 h; acute, from 24 h to 7 days; and late, more than 7 days.

Each subsection describes the biochemical markers that have shown statistically significant results in various studies and that have been comparable with each other because their concentration results are indicated with numerical values and expressed in pg/µL, in a concentration unit that can be converted to this through the standardisation of units or in mRNA expression. The baseline values represent mean concentrations across multiple studies. Only baseline values with the same collection and analysis methodology were used, due to the difference in the sample lengths between the studies, which means they only coincide at the starting point.

The biochemical markers are listed in order of relevance in the scientific literature. Such quantitative data are essential not only for clinical interpretation but also for determining how biomaterials influence bone metabolism in orthodontic contexts.

### 3.1. Bone Formation Markers

Bone formation markers reflect the activity of osteoblasts at different stages of differentiation and in synthesis, maturation and mineralisation of osteoid.

#### 3.1.1. Osteoprotegerin (OPG)

Soluble protein released by immature OBs, composed of 380 amino acids. Its main function is to inhibit osteoclastic activity by binding to RANKL, promoting OCs apoptosis and regulating osteoclastogenesis. Its concentration decreases with age. Its presence in orthodontic movement has been documented, reinforcing its clinical relevance [35,36,38,39,41,45,47,48].

It acts as a marker of bone formation because, although it does not directly stimulate bone formation, its action promotes an environment conducive to osteogenesis in acute phases, increasing in areas of pressure and decreasing in areas of tension.

#### 3.1.2. Transforming Growth Factor β1 (TGF-β1)

Multifunctional cytokine that regulates bone formation. It is the most abundant isoform in the bone matrix and is activated during its degradation by OCs. It promotes the migration and differentiation of mesenchymal cells into OBs, stimulates extracellular matrix synthesis, and exerts anti-inflammatory effects. In the presence of RANKL and M-CSF, it can also promote osteoclastogenesis, demonstrating the complexity of its action [36,38].

Under normal conditions, it behaves as a marker of bone formation, with increased expression in areas of tension and decreased expression in areas of pressure during the acute phases of orthodontic tooth movement.

#### 3.1.3. Interleukin 27 (Il-27)

Anti-inflammatory cytokine produced in antigen-presenting cells. It modulates T cell responses, with an inhibitory effect on bone resorption, as it correlates negatively with RANKL [42].

It is considered a marker of bone formation, with a transient role in the regulation of remodelling. Its expression increases in the early phases of orthodontic force-induced bone remodelling.

#### 3.1.4. Interleukin 10 (IL-10)

Cytokine with anti-inflammatory functions that regulates the immune response. The anti-inflammatory phase of an inflammatory process begins with the secretion of IL-10 and certain growth factors, leading to the activation and proliferation of fibroblasts, which are essential for tissue repair. In addition, it inhibits the production of pro-inflammatory cytokines such as TNF-α, contributing to the restoration of immune homeostasis [51].

It acts as a marker of bone formation in acute phases, with increased expression in areas of tension during the initial phases of orthodontic tooth movement [38].

#### 3.1.5. Osteocalcin (OCN)

Non-collagenous protein most abundant in the bone matrix, secreted by OB. It binds to hydroxyapatite and participates in bone remodelling and maintenance [52,53,54].

It is considered a marker of bone formation, due to its statistically significant increase on the pressure side during the acute phases.

#### 3.1.6. Type 1 Collagen (COL-1)

Protein produced by OBs that forms part of the extracellular matrix. Its expression is associated with the anabolic activity of the PDL during tooth movement induced by orthodontic forces.

It acts as a marker of bone formation, similar to IL-10 and OCN, with increased expression in areas of tension during the acute phases of orthodontic tooth movement [38].

Information about the baseline values for each biochemical marker of bone formation, together with their variation over time, both on the compression side and on the tension side, is summarised in Table 3.

### 3.2. Bone Resorption Markers

Bone resorption markers reflect OC activity and the degradation of the mineralised bone matrix.

#### 3.2.1. Receptor Activator of Factor Nuclear Kappa β Ligand (RANKL)

Pro-osteoclastic cytokine of the TNF family that activates OCs differentiation, function, and survival by binding to RANK. It is correlated with some interleukins [36,38,39,41,42,48,55].

In addition, Kawasaki’s study [45] showed that there is an age-related variable: adults show higher RANKL than adolescents in pressure areas.

This is why RANKL is considered a marker of bone resorption with increased expression in pressure areas during the acute phases of orthodontic movement.

#### 3.2.2. Tumour Necrosis Factor (TNF-α)

A proinflammatory cytokine produced by immune cells such as macrophages that promotes osteoclastogenesis, stimulates innate immunity and stimulates other mediators such as certain interleukins, converting membrane RANKL into soluble form [32,39,40,42,47].

It is considered a marker of bone resorption, with a role in the acute phases of orthodontic force-induced bone remodelling, as it is one of the first to be activated after injury. Its expression is higher in areas of pressure than in areas of tension.

#### 3.2.3. Interleukins

Interleukin 1β (IL-1β) is a proinflammatory cytokine secreted by mast cells in response to the detection of microorganisms. Its main function is to stimulate osteoclast activity and survival, initiating the proinflammatory phase of bone remodelling. This process promotes osteoclastogenesis, facilitating the destruction of osteoid and mineralised matrix. In the context of orthodontic movement, IL-1β has been identified as a key mediator in the initial inflammatory response following the application of mechanical forces. Its elevated presence may indicate intense osteoclast activation, associated with inflammatory processes or an exaggerated response to orthodontic treatment. It is also associated with pain perception [34,36,39,44,47]. IL-1β behaves as a key marker of bone resorption in the acute phase of orthodontic movement, with increased expression in areas of pressure.

Interleukin 6 (IL-6) is a proinflammatory cytokine with multiple immunological and haematopoietic functions. It stimulates the production of neutrophils and proteins in the bone marrow, reflecting its active role in the immune response. In bone tissue, it facilitates osteoclast-mediated bone resorption, stimulating both its formation, differentiation and resorptive activity [32,34,39,46]. IL-6 acts as a marker of bone resorption in acute phases during orthodontic movement due to its rapid involvement in bone remodelling induced by external stimuli, increasing its expression in areas of pressure.

Interleukin 17A (IL-17A) is a proinflammatory and osteoresorptive cytokine that promotes direct osteoclastogenesis and RANKL pathway. In addition, there is a positive correlation between this interleukin and RANKL [42]. It acts as a marker of bone resorption with greater expression in the acute and late phases of orthodontic movement, present on both the pressure side and the tension side in bone regeneration.

Interleukin 17F (IL-17F) is a proinflammatory and osteoresorptive cytokine that promotes osteoclastic differentiation. Its pattern of evolution is similar to that of IL-17A [46]. It is considered a marker of bone resorption with involvement in the acute and late phase of bone remodelling, in parallel with IL-17A, with an increase in its concentration on both the pressure side and the tension side.

Interleukin 23 (IL-23) is a proinflammatory cytokine that stimulates the differentiation of Th17 lymphocytes, thereby regulating osteoclast activation. There is a positive correlation between this mediator and RANKL [42]. It is a marker of bone resorption present in the acute and late phases of bone remodelling on the pressure side and on the tension side.

#### 3.2.4. Osteopontin (OPN)

Proinflammatory protein with the ability to promote OC adhesion to the bone matrix, a key event in bone tissue degradation. It facilitates interaction between OCs and the mineralised substrate, allowing efficient resorption. Its evolution is parallel to that of RANKL. In addition, it is synthesised by OBs, which demonstrates the functional interrelationship between bone-forming and bone-resorbing cells. This functional duality makes it an interesting marker for studying the dynamic balance of bone remodelling during orthodontic treatment [35,36].

It is a marker of bone resorption, whose concentration is stable in areas of tension and increased in areas of pressure during the late phases of bone regeneration induced by orthodontic movements.

#### 3.2.5. Prostaglandin E2 (PGE2)

Inflammatory mediator involved in osteoclast activation and subsequent bone tissue degradation. In addition, its secretion is associated with pain perception by sensitising nerve cells that detect tissue damage [37].

Because of this, it acts as a marker of acute bone resorption, increasing its expression in areas of pressure more than in areas of tension.

#### 3.2.6. Receptor Activator of Factor Nuclear Kappa β (RANK)

RANKL receptor, composed of 317 aminoacids, located in the osteoblastic membrane. Its activation induces the formation, differentiation, and activation of OC, thus being an essential factor in their life cycle [35].

It is considered a marker of bone resorption, as it shows increased expression in the acute phases of bone regeneration induced by orthodontic movements, especially in areas of pressure.

#### 3.2.7. Calcitonin (CT)

Peptide hormone with antiresorptive and analgesic activity. It inhibits osteoclastic activity and is released in response to increased Ca^2+^ during bone resorption. Furthermore, its expression is inversely associated with pain intensity, suggesting a possible modulatory effect on neurogenic inflammation [33].

It is considered a marker of bone resorption in late phases, with increased concentration on the compression side. However, no significant changes in its concentration were observed in tension areas.

#### 3.2.8. Matrix Metallopeptidases

Matrix Metallopeptidase 1 (MMP-1) is a protease with osteoclastic activity and collagen matrix degradation function along with other extracellular matrix proteins, an essential process for bone regulation [37]. It acts as a marker of bone resorption in late phases with increased expression in areas of pressure and decreased expression in areas of tension during orthodontic movement.

Matrix Metallopeptidase 9 (MMP-9) is a protease similar to MMP-1 with a fundamental role in the degradation of extracellular matrix components during osteoclastic activation, such as collagen, playing a crucial role in bone remodelling [39]. It is considered a marker of bone resorption in late phases, with increased expression in areas of tension and pressure during orthodontic movement, although more noticeably in the latter.

#### 3.2.9. Substance P (SP)

Neuropeptide belonging to the tachykinin family that stimulates osteoclastic activity and is associated with neurogenic inflammation by acting as a neuromodulator and neurotransmitter. It also takes part in the transmission and perception of pain, inflammation, and stress response [35].

It behaves as a marker of bone resorption with increased expression in areas of pressure and tension, although to a greater extent on the pressure side. Its role appears to be limited to the acute phase of orthodontic movement.

To understand and complete the necessary information regarding biochemical markers of bone resorption, Table 4 is attached below, which details the baseline values for each biochemical marker of bone resorption with its standard deviation and variation over time, both on the pressure side and on the tension side.

In order to facilitate understanding and summarising the role of biochemical markers in their different phases, the following Table 5 is included.

## 4. Discussion

There are numerous biochemical markers that are currently intended to be used to provide information and even accelerate scaffold-base bone regeneration processes in patients undergoing orthodontic treatment in a safe and controlled manner in order to provide more effective and personalised treatment and to be able to evaluate the biological performance of functional biomaterials potentially created for this purpose.

The study of biochemical markers in the context of orthodontic treatment allows for a deeper understanding of the molecular mechanisms that regulate scaffold-based bone remodelling. The interaction between OBs and OCs is mediated by a complex network of biochemical signals, many of which are altered by the application of mechanical forces. In this sense, the markers analysed in this study offer a detailed view of the processes of bone formation and resorption, as well as the inflammatory and reparative phases that accompany tooth movement, reaffirming their importance as key tools for evaluating how potential functional biomaterials influence these processes, since they allow for the objective measurement of the biological response induced by materials designed to modulate cellular activity.

The results in Table 1 and Table 2 show the biochemical markers found in bone formation and resorption processes, respectively, and how they behave on the pressure side and the tension side. The baseline values are calculated based on the mean of the studies that reported numerical data whose unit is pg/µL or one convertible to this, with measurements in the GFC and with a distinction between the pressure side and the tension side. This led to the exclusion of numerous studies due to the lack of a common criterion for their inclusion. Furthermore, when calculating the means and standard deviations of the studies that met these requirements, these data may be slightly higher or lower than those found individually in each study.

The variability in reference values and measurement methods for these markers raises the need to establish specific common parameters. Factors such as age, sex, hormonal status, the presence of systemic pathologies, and even sample collection and the technicians responsible for performing this work can influence the interpretation of the results. It is therefore recommended that each analysis be contextualised within the clinical profile of the patient and the study itself.

Table 1 includes the biochemical markers of bone formation OPG, TFG-β1, and IL-27. Only these are included due to the absence of studies with statistically significant data on the other biochemical markers that could potentially be present in bone formation. We thus highlight the specificity and rigour of these biomarkers.

The OPG results are relatively similar in the included studies, with slight discrepancies at 24 h, probably due to the absence of a common criterion for selecting the time points evaluated. The limitations of the studies must be considered, as the studies by Kawasaki [41] and Toygar [47] only described comparable numerical data with statistically significant results on the compression side and did not consider the tension side. In addition, some studies, such as those by Flórez-Moreno [54] and Rody [56], were discarded because they evaluated saliva instead of GCF, thus making it impossible to discern between the pressure side and the tension side.

No contradictions were found between the included studies about the other two biochemical markers of bone formation included in the study, TGF-β1 and IL-27, probably due to the limited sample size and the lack of in-depth evaluation of follow-up over time.

Current evidence of IL-10, OCN and COL-1 is less definitive due to the limited availability of comparable measurements that can easily be compared with the other biochemical markers present in the review. For this reason, they are not yet considered reliable markers: further research is recommended to determine their effectiveness [1,38,53].

There are other biochemical markers that have not been included in this study because they do not meet the necessary criteria, which we will discuss below.

Metallopeptidase Inhibitor 1 (TIMP-1) and Metallopeptidase Inhibitor 2 (TIMP-2) are tissue inhibitors of metalloproteinases that play key roles in regulating extracellular matrix remodelling during orthodontic tooth movement. Both act by blocking the activity of matrix metalloproteinases, which degrade structural components of periodontal tissue. Although the studies by Garlet [38] and Grant [39] investigate this marker on the tension and pressure sides, they do not provide baseline numerical values and values at different time points, so they are not considered markers of choice and are therefore not included in Table 2.

Alkaline phosphatase (ALP) is a non-specific enzyme produced by OBs, present in bone, liver and the digestive system. It is responsible for the dephosphorylation of organic compounds and increased bone mineralisation. Elevated levels are associated with diseases such as Paget’s disease, while decreased levels may indicate osteoporosis [18,57,58,59]. There are no studies with statistically significant results that meet the inclusion criteria for this biomarker.

Finally, parathyroid hormone (PTH) is a hypercalcaemic hormone produced by the parathyroid glands. It regulates Ca^2+^ and P in the blood through rapid (minutes) and slow (weeks) phases, the latter associated with OCs proliferation. Its secretion increases when extracellular Ca^2+^ decreases [2,13,15,60,61]. As with the previous marker, no studies dealing with PTH have been included due to the absence of comparable parameters.

Table 3 includes the biochemical markers of bone resorption RANK-L, TNF-α, IL-1β, IL-6, OPN, PGE2, RANK, CT, IL-17A, IL-17F, IL-23, MMP-1, MMP-9 and SP. Below we describe the problems and contradictions that were found.

With regard to RANKL, despite the similarities in results between studies and the statistically significant differences between the increase in RANKL concentration on the pressure side and its decrease on the tension side, Tuncer’s study [48] found no significant differences between these areas over time. This may be due to the type of force and the age of the patient: Otero [45] shows a correlation between the increase in this marker and the magnitude of force. There are also slight differences in the recovery of baseline RANKL values between studies, probably due to the difference in the timing of sampling, ranging from 7 to 21 days and even 12 weeks in the case of Lin’s study [42]. Kawasaki’s study [41] is limited in that it performed the analysis and provided data only on the compression side. Furthermore, Flórez-Moreno’s study [54] is not included in this review because the peak is found 8 weeks after the intervention, a value that is considered altered, probably due to the inaccuracy of the sample collection site, as the samples are collected in saliva.

Studies that include TNF-α agree with each other in their general pattern over time, although they show slight discrepancies in the timing of the peak and return to baseline values. Of particular note is Padisar’s study [46], which, despite agreeing with the other articles on the increase and decrease in the concentration of this marker, found no statistically significant differences on either side. Alikhani’s study [37] was only considered on the pressure side, as it does not show data on the tension side. There are temporal limitations, such as in Garlet’s study [38], which, in addition to not providing numerical data to find baseline values, only evaluate both cases 7 days after the intervention, without prior or subsequent follow-up. Furthermore, there are studies that have not been included in Table 3, such as Ren’s [62], due to the absence of numerical data comparable with the rest of the studies and the discordance of results in the pressure zone (the only one it determines), as it considers that this marker increases up to 3 months of evolution, decreasing slightly at 4 months, still far from the baseline values. This can be explained by the small sample size evaluated and the design of the study itself, in which elastic separators were used, a method that is much less replicable in terms of the exact force applied between patients.

All studies agree that IL-1β increases in pressure areas, where bone resorption is activated. Most report a decrease around day 7, indicating resolution of the inflammatory phase. However, Grant [39] and Castrofolio [36] describe an expression that is still elevated at that time. These results may be due to several factors. In Grant’s study [39], the force was applied using stainless steel, which is a very rigid metal that can cause more abrupt movements and, therefore, require a greater force than expected. Conversely, Castrofolio’s study [36] used Invisalign^®^ to apply force to the tooth surfaces, so these results may differ due to the different method employed.

Atuğ Özcan’s study [63] has not been included due to the time points it takes (1 month and 6 months), which are very far apart from the rest of the studies, and because it only shows statistically significant results at 6 months, which contradicts all other authors, possibly due to a different sample collection method in saliva and CF without specifying the tooth surface compared to the other studies. The studies by Alikhani [34] and Luppanapornlarp [44] only showed results on the compression side. The studies by Gujar [64] and Gameiro [65] did not distinguish between pressure and tension, which limits the interpretation of their results due to the medium’s nonspecificity, as they do not coincide with the rest of the studies that do differentiate between these two areas and, therefore, are not included in this review.

Studies examining IL-6 agree that it increases in pressure areas, where inflammation and bone resorption are activated. Most report a decrease around day 7, although Grant [39] describes an expression that remains elevated, probably for the same reasons as IL-1β. Alikhani’s study [34] was only considered on the pressure side, as it does not provide data on the tension side. It should be noted that the studies by MacLaine [66], Ren [62] and Reiss [67] did not find statistically significant results for this marker at any of the points evaluated; however, none of these studies were included in the review. MacLaine’s study [66] was not included because the samples were collected through blood analysis. In regard to Ren’s study [62], it was due to the difference in study design compared to the rest. Finally, Reiss’s study [67] was conducted on saliva and not on GCF.

Interleukins are low molecular weight inflammatory mediators secreted by cells such as monocytes, macrophages, fibroblasts, lymphocytes and osteoblasts, which regulate the immune response and periodontal remodelling induced by orthodontic forces. Interleukin 10 (IL-10), Interleukin 2 (IL-2), Interleukin 8 (IL-8), Interleukin 13 (IL-13) and Interleukin 16 (IL-16) should be considered as possible biochemical markers of bone resorption as they participate in different phases of tooth movement. Studies are beginning to review them for this purpose, although there are still no studies with common parameters to discern between their specificity and reliability. The most advanced of these is Alikhani’s study; however, the interleukins they considered (IL-1a and IL-8) have not been included because he only deals with the compression side, and there are no further studies to complete this information.

With regard to OPN, Castrofolio [36] found a significant increase on the tension side at 3 weeks, a finding that cannot be countered by Barbieri [35], as he has a sample taken at 7 days, which is too short a time to see the real evolution of this marker.

PGE2 does not show significant differences between studies. Gameiro’s study [65] was not included due to the lack of data comparable with the other studies, as it does not distinguish between the pressure side and the tension side. However, it should be noted that there were no significant differences with regard to the test and control of this marker. This may be explained by the small sample size and the study design.

Despite finding a greater number of specific and reliable biochemical markers in bone resorption than in bone formation, we include below those that are considered to be the subject of study for potential complementary use in future bone resorption analyses.

Tartrate-resistant acid phosphatase (TRAP) is an enzyme belonging to the acid phosphatase group and is the only isoform that is not inhibited by L-tartrate. It contributes to the hydrolysis of phosphomonoesters at acidic pH and is found in bone as a tartrate-resistant bone isoenzyme. Its increase indicates greater osteoclastic activity, associated with pathologies such as Paget’s disease. Although its use has declined, isoenzyme 5b, known as tartrate-resistant acid phosphatase 5b (TRAP5b), continues to be studied for its potential as a marker of bone resorption. This isoenzyme has not been included in this review because the only study that reports it, Karaduman’s study [40], does not provide baseline values or comparable numerical data for inclusion in Table 3.

In addition to these potential biochemical markers of bone resorption, urinary calcium and hydroxyproline have been the focus of numerous articles for years.

Urinary calcium is an easy-to-observe marker that can confirm osteoclastic activity. However, its specificity is low due to possible hormonal and dietary variations in the patient. Although useful as a general indicator, it is not considered a reliable marker of bone resorption in specific clinical contexts and has therefore not been included in the study.

Hydroxyproline is a non-essential amino acid derived from the hydroxylation of proline, 90% of which is released during the degradation of bone collagen. Present in bones and connective tissue, it contributes to collagen synthesis and protection against microorganisms. It was the first bone resorption marker to be identified, but its low specificity—due to dietary and hepatic influences and the synthesis of new collagen—has limited its clinical use. It has now been replaced by more accurate markers.

## 5. Limitations and Future Research Directions

Although the sample size in some studies is small, the markers analysed have been previously validated in the literature as indicators of bone remodelling. In this article, the consistency of the changes observed at different times during tooth movement supports the specificity and reliability of these markers. However, these findings should be interpreted as preliminary trends, and further studies with a larger sample size are required to confirm and generalise the results.

A comprehensive evaluation of these potential markers, which have not yet been investigated in the field of orthodontics, will allow us to identify which could be useful as an additional diagnostic complement to those whose efficacy has already been proven. Similarly, their analysis opens up the possibility of assessing how these biomarkers can be used to measure the interaction and performance of functional biomaterials applied in orthodontics, providing objective information on their ability to modulate bone remodelling.

Furthermore, more research is needed, focusing on both the markers considered in this review and those mentioned in the previous section, which were excluded from this study because they did not meet the necessary criteria. These studies should use standardised criteria for sample collection and analysis and have larger sample sizes to allow comparisons at the same time points for each marker. This would help their integration into orthodontic practice, contributing to personalised and accelerated treatment, the early detection of potential complications, the establishment of common parameters for evaluating scaffolds and functional biomaterials and consequently, therapeutic efficiency would be improved.

In order to specifically evaluate a functional biomaterial using the biochemical markers, certain prerequisites must be met. A clear definition of the material’s aim must be specified, focusing on its target mechanisms (in this case, bone formation or resorption). A standardised statistical plan and success criteria must be defined before the experimental phase begins (mechanical efficacy, understood as adequate tooth displacement without adverse effects, and biological efficacy, depending on the profile of the biochemical marker). The selection of the biochemical marker must be aligned with the aim, considering its composition and release, its intrinsic bioactivity and its safety, and considering the temporal phase under study. As previously mentioned, it must be specific to bone metabolism, accessible by non-invasive methods, and allow for reliable and reproducible interpretation. Once this has been performed, the experimental design must be conducted with controls and predefined timelines, and the validation must be conducted using a reliable technique (ELISA is recommended), with standardised units (ideally pg/µL) and a method that allows for reproducibility.

## 6. Conclusions

The analysis of biochemical markers involved in bone remodelling during orthodontic treatment provides a valuable tool for understanding the cellular and molecular processes that regulate the response of bone tissue to mechanical stimuli. The differentiation between markers of bone formation and resorption allows research into the acceleration of orthodontic movement, accurate identification of the phases of remodelling, evaluation of its progression, detecting potential alterations, and understanding the functional scaffolds and biomaterials that interact with bone subjected to orthodontic forces.

The most scientifically relevant markers recommended for the study of bone formation are, in this order, OPG, TGF-β1 and IL-27, which reflect the regenerative capacity of bone and its anabolic response to treatment.

Similarly, the most scientifically relevant markers of bone resorption are, in this order, RANK-L, TNF-α, IL-1β, IL-6, OPN, PGE2, RANK, CT, IL-17A, IL-17F, IL-23, MMP-1, MMP-9 and SP, which allow for monitoring of osteoclastic activity and the magnitude of orthodontically induced destruction.

The presence of these biomarkers, together with their accessibility through non-invasive techniques, makes them ideal candidates for clinical monitoring. However, their interpretation must be conducted with caution, considering interindividual variability and external factors that may influence their levels.

Further studies are needed to consolidate the reliability of the biochemical markers analysed in this study and to identify others for which scientific evidence is still limited. This work is essential to validate their application as diagnostic and prognostic tools in orthodontics through the use of scaffolds and functional biomaterials, with the aim of optimising, personalising and accelerating clinical treatment.

## Figures and Tables

**Table 1:** Assessment of the quality of studies through Methodological Index for Non-Randomised Studies (MINORS).

Study	Clearly Stated Aim	Consecutive Patients	Prospective Collection Data	Endpoints	Assessment Endpoint	Follow-Up Period	Loss Less Than 5%	Study Size	Adequate Control Group	Contemporary Group	Baseline Control	Statistical Analyses	Minors
**Afacan B, 2019 [32]**	2	2	2	2	2	2	0	2	2	2	2	2	22
**Alarcón JA, 2013 [33]**	2	0	2	2	0	2	0	2	2	2	2	2	18
**Alikhani M, 2013 [34]**	2	2	2	2	2	2	2	2	2	1	2	2	23
**Barbieri G, 2013 [35]**	2	2	2	2	0	1	0	2	2	2	2	2	19
**Castrofolio T, 2017 [36]**	2	2	2	2	0	2	0	2	2	2	2	2	20
**Dudic A, 2006 [37]**	2	0	2	1	0	1	0	2	2	2	2	2	16
**Garlet TP, 2007 [38]**	2	0	2	0	0	0	0	1	2	2	2	2	13
**Grant M, 2013 [39]**	2	0	2	2	0	2	2	2	2	2	2	2	20
**Karaduman B, 2015 [40]**	2	0	2	2	0	2	0	1	2	2	2	2	17
**Kawasaki K, 2006 [41]**	2	0	2	2	0	1	0	2	2	1	2	2	16
**Lin T, 2020 [42]**	2	0	2	2	0	2	0	2	2	2	2	2	18
**Lee KJ, 2004 [43]**	2	0	2	2	0	2	0	2	2	2	2	2	18
**Luppanapornlarp S, 2010 [44]**	2	0	2	2	0	2	0	2	2	2	2	2	18
**Otero L, 2016 [45]**	2	0	2	2	0	1	0	2	2	2	2	2	17
**Padisar P, 2018 [46]**	2	0	2	2	0	2	0	2	2	2	2	2	18
**Toygar HU, 2008 [47]**	2	2	2	2	0	2	0	2	2	1	2	2	19
**Tuncer BB, 2013 [48]**	2	0	2	2	0	2	0	1	2	2	2	2	17

**Table 2:** Information from studies that evaluated biochemical markers during orthodontic movements.

Study	Biochemical Markers	Follow-Up	N	Teeth	Intervention	Treatment Methods	Sample Collection	Analysis Methods	Outcomes
Afacan B, 2019 [32]	G-CSF, GM-CSF, IFN-α., IFN-γ, IL-1β, IL-1RA, IL-2, IL-2R, IL-4, IL-5, IL-6, IL-7, IL-8, IL-10, IL-12, IL-13, IL-15, IL-1, TNF-α.	0 24 h 28 days	15	Maxillary canines.	Following extraction of the maxillary first premolars and placement of two MTs, the upper canines were randomly distalised with a continuous force of 75 or 150 g.	Preadjusted edgewise orthodontic brackets (Gemini; 3M Unitek, Monrovia, CA, USA) with 0.022″ slots and 0.019″ × 0.025″ segmented arches.	Paper strips (PerioPaper; OraFlow Inc., Plainview, NY, USA).	Luminex.	In the first 24 h, IL-8 and MCP-1 levels were lower on the pressure sides that received more force. At 28 days, TNF-α and IL-1RA levels increased on both sides.
Alarcón JA, 2013 [33]	CT.	0 1 h 24 h 7 days 15 days	15	First upper premolars.	Brackets were placed on the upper central incisors, and an orthodontic force of 100 g was applied using a pre-stretched elastic chain to close the diastema. No archwire was used.	Brackets (0.022–0.028 in. Mini Master, American Orthodontics, Sheboygan, WI, USA) and elastomeric chain (Memory chain, American Orthodontics, Sheboygan, WI, USA).	Periopaper strips (Harco, Tustin, CA, USA).	Western Blot.	CT levels increased significantly at the compression site, especially between 1 h and 7 days later.
Alikhani M, 2013 [34]	CCL-2 (MCP1), CCL-3, CCL-5 (RANTES), IL-8 (CXCL8), IL-1α, IL-1β, IL-6, TNF-α.	0 1 day 7 days 28 days	20	Maxillary canines.	Following extraction of the first premolars, the canines were retracted using calibrated 100 g NiTi closing-coil springs connected from a temporary anchorage device to a power arm on the canine bracket.	Preadjusted edgewise orthodontic brackets (Gemini; 3M Unitek, Monrovia, CA, USA) with 0.022-inch slots and segmented 0.019 × 0.025-inch stainless steel arch wires.	Filter-paper strips (Oraflow, Smithtown, NY, USA).	Glass slide-based protein array.	IL-1α, IL-1β, TNF-α, and IL-6 increased by 4.6, 2.4, 2.3, and 1.9, respectively, in the control group (compression side), and by 8.6, 8.0, 4.3, and 2.9, respectively, in the experimental group.
Barbieri G, 2013 [35]	RANK, OPG, OPN, TGF-β1.	0 24 h 7 days	10	First molars.	An orthodontic elastic separator was placed on the mesial side of the molars.	Orthodontic elastics.	Paper strips (Harco, Tustin, CA, USA).	ELISA.	Both increased expression of bone resorptive mediators (RANK and TGF-β1) and decreased expression of a bone-forming mediator (OPG) on the compression side were detected.
Castrofolio T, 2017 [36]	IL-1β, RANKL, OPG, OPN, TGF-β1.	0 1 h 7 days 21 days	10	Second upper molars.	Distalisation of upper second molars by 0.25 mm using invisible orthodontics.	Invisalign^®^ (Align Technology, San Jose, CA, USA) orthodontic appliance.	Paper strips (Oraflow Inc., Plainview, NY, USA).	ELISA.	An increased concentration of bone modelling and remodelling mediators at the presure (IL-1β, RANKL) and tension sites (TGF-16, OPN) was observed.
Dudic A, 2006 [37]	IL-1β, SP, PGE2.	−7 days 0 1 min 1 h 1 day 7 days	18	First upper or lower molars.	Orthodontic elastic separators were inserted in the mesial area of the upper or lower first molars.	Orthodontic elastic separators.	Durapore filter membranes (pore size: 0.22 μm; Millipore, Bedford, MA, USA).	ELISA.	The mean IL-1β, SP, and PGE2 values were significantly higher in the E-M sites compared with the C-M sites, on all occasions.
Garlet TP, 2007 [38]	COL-I, TIMP-1, TNF-α, RANKL, MMP-1, IL-10, OPG, OCN, TGF-ß.	7 days	7	Upper first premolars.	Teeth were exposed to orthodontic forces (7 N).	The rapid maxillary expansion procedure was performed with Hyrax appliances (Dentaurum, Germany).	NR	qPCR.	The compression side exhibited higher expression of TNF-α, RANKL, and MMP-1, whereas the tension side presented higher expression of IL-10, TIMP-1, COL-I, OPG, and OCN.
Grant M, 2013 [39]	MMP9, TIMPs., RANKL, OPG.	0 4 h 7 days 42 days	20	GE: Maxillary canines. GC: Maxillary second molars.	After extraction of the upper first premolars, the canines were distalised using brackets with a force of 100 g.	MBT brackets (3M Unitek, Berkshire, UK), with the following archwire sequence: 0.014″ Ni-Ti → 0.018″ Ni-Ti → 0.018” stainless steel.	Paper strips (Periopaper™ strips (Oraflow Inc., Smithtown, NY, USA)).	Luminex.	Tension sites showed significant increases in IL-1β, IL-8, TNFα, MMP-9 and TIMPs 1 and 2 across all time points, while compression sites exhibited increases in IL-1β and IL-8 after 4 h, MMP-9 after 7 and 42 days and RANKL after 42 days.
Karaduman B, 2015 [40]	TNF-α, IL-10, TRAP.	0 1 h 24 h 7 days 28 days	9	Canines.	After extraction of the upper first premolars, the upper canines were distalised using brackets with a continuous force of 150 g.	Orthodontic brackets (Omni Roth, GAC International, Inc., Bohemia, NY, USA) with 0.016 × 0.022” Sentalloy segmented arches.	Paper strips (Periopaper-ProFlow Inc., Amityville, Nueva York).	ELISA.	TNF-aα and TRAP5b levels in distal and mesial sites of the test teeth were significantly higher than that at both sites of the controls. The IL-10 concentration decreased during experimental period at both sites of the control and test teeth.
Kawasaki K, 2006 [41]	RANKL, OPG.	0 1 h 24 h 168 h	30	Maxillary canines.	After extraction of the first upper premolars, the canines were distalised using brackets with an initial force of 250 g.	Edgewise brackets with 0.018 × 0.025″ slot (Tomy International Inc., Tokyo, Japan).	Paper strips (Periopaper; Harco, Tustin, California).	ELISA.	After 24 h RANKL levels were increased and those of OPG decreased from the compression side.
Lin T, 2020 [42]	IL-17, IL-23, IL-27, RANKL.	0 1 h 24 h 1 week 4 weeks 12 weeks	30	Maxillary canines.	After extraction of the first maxillary premolar, the maxillary canines were distalised using fixed orthodontic treatment with a force of 150 g.	NR	Paper strips (Periopaper, Interstate Drug Exchange, Amityville, NY, USA).	ELISA.	IL-17A, IL-17F, IL-23 and RANKL increased significantly in the first week. IL-27 decreased significantly in this period. IL-17A, IL-17F and IL-23 were positively correlated with RANKL. IL-27 was negatively correlated with RANKL.
Lee KJ, 2004 [43]	IL-1β, PGE2.	0 1 h 24 h 1 week 1 week and 1 h 2 weeks 2 weeks and 1 h 2 weeks aand 24 h 3 weeks	10	Maxillary canines.	After extraction of the first maxillary premolars, the canines were distalised using continuous force and weekly interrupted force.	Ni-Ti spring calibrated to 100 g and retractor with expansion screw, reactivated weekly.	Paper strips (Periopaper; Proflow, Amityville, NY).	ELISA.	Both types of force (continuous and intermittent) caused a significant increase in IL-1β and PGE2 at 24 h.
Luppanapornlarp S, 2010 [44]	Il-1β.	0 1 h 24 h 1 week 1 month 2 months	16	Maxillary canines.	After extraction of the first premolars, the upper canines were distalised by applying a continuous force of 50 g or 150 g.	0.022-inch slot brackets (Ormco Corp., Brea, CA, USA) and 0.018 × 0.025-inch segmented stainless steel wire arches.	Paper strips (Periopaper; Proflow™ Incorporated, Amityville, Nueva York).	ELISA.	IL-1ß concentration in the 150 g group showed the highest level in the compress side at 24 h and 2 months with significant differences compared with the control group.
Otero L, 2016 [45]	RANKL, OPG.	7 days	32	Upper first premolars.	Fixed orthodontic treatment using brackets on the upper first premolar loaded with a force of 4 oz (113 g) or 7 oz (198 g). After this, the tooth was extracted.	Edgewise brackets.	Curette (Hu-Friedy).	ELISA.	RANKL protein level was significantly greater in the pressure sides with 7 oz. Changes in RANKL/OPG protein ratio in experimental and control groups showed a statistically significant difference. OPG did not show statistically significant association with any group.
Padisar P, 2018 [46]	TNF-α, IL-6.	0 1 h 28 days	10	Maxillary canines.	After extraction of the first premolars, the upper canines were distalised using brackets and a force of 150 g.	0.022-inch edgewise brackets and a 0.017 × 0.025-inch arch.	Paper strips (Oraflow, Nueva York).	ELISA.	The level of IL-6 and TNFα at both sides of study teeth was higher than both sides of control teeth at 1 h but the difference was not statistically significant.
Toygar HU, 2008 [47]	OPG.	0 1 h 24 h 168 h 1 month 3 months	12	Canines.	After extraction of the first premolars, the canines were distalised using brackets and a continuous force of 150 g.	Vertical slot brackets (GAC International Inc., Bohemia, NY) together with a 0.016 × 0.022” segmented stainless steel archwire.	Paper strips (Periopaper, Harco, Tustin, California).	ELISA.	OPG concentrations in compression sites of the test teeth were decreased in a time-dependent manner compared with the baseline measurements.
Tuncer BB, 2013 [48]	OPG, RANKL	0 1 h 24 h 168 h 1 month	9	Maxillary canines.	Distalisation forces were applied generating a continuous force of 200 g.	Orthodontic brackets with segmental 0.016 × 0.022”, stainless-steel archwires and sentalloy closed coil springs.	Paper strips (Periopaper-ProFlow Inc.)	ELISA.	OPG concentration showed an increase on the tension side, while there was a decrease on the compression side at 1 h. RANKL expression did not show significant changes.

NR = Not Reported.

**Table 3:** Biochemical markers of bone formation.

Biochemical Marker	Baseline Value	Pressure Side	Tension Side
OPG	68.22 ± 24.6	Increases from 1 h to 24 h and decreases at 7 days.	Decreases after 1 h with partial recovery at 24 h and return to baseline at 21 days.
TFG-β1	173.43 ± 155	Increases from 6 to 24 h, peaking at 7 days.	Decreases slightly after 24 h and remains stable at 7 days.
IL-27	0.338 ± 0.069	Increases from baseline to the first hour, decreasing from 24 h to 4 weeks, with a return to baseline at 12 weeks.	Increases from baseline at 1 h, decreasing from 24 h to 4 weeks, with a return to baseline at 12 weeks.
IL-10	0.29 ± 0.76 ^1^	Increases from baseline, remaining steady until 7 days.	No significant changes.
OCN	0.16 ± 0.94 ^1^	Increases from baseline, remaining steady until 7 days.	Increases slightly at 7 days.
COL-1	1.7 ± 1.1 ^1^	Increases from baseline, remaining steady until 7 days.	Increases slightly at 7 days.

^1^ These biochemical markers are expressed in mRNA expression and not pg/µL.

**Table 4:** Biochemical markers of bone resorption.

Biochemical Marker	Baseline Value	Pressure Side	Tension Side
RANKL	0.0012 ± 0.0004	Increases after 1 h, peaking after 7 days, and then decreases to 12 weeks.	Decreases at 1 h to 7–21 days.
TNF-α	3.62 ± 3.63	Increases after 4 h, peaking after 24–72 h, remaining steady until 7 days, and then decreasing after 14–28 days.	Increases slightly at 1 h, returning to baseline at 28 days.
IL-1β	36.4 ± 13.2	Increases from 4 h to 7 days, at which point it begins to decrease.	Increases slightly at 24 h and decreases at 7 days.
IL-6	2.56 ± 1.84	Increases from 4 h with a peak at 24 h, sustained until 7 days and returning to baseline at 28 days.	Increases slightly at 1–4 h, returning to baseline at 28 days.
IL-17A	24.75 ± 7.65	Increases from 24 h and remains stable at 4 weeks, returning to baseline at 12 weeks.	Increases from 24 h and remains at 4 weeks, returning to baseline at 12 weeks.
IL-17F	0.0486 ± 0.0163	Increases from 24 h and remains stable at 4 weeks, returning to baseline at 12 weeks.	Increases from 24 h and remains stable at 4 weeks, returning to baseline at 12 weeks.
1L-23	0.062 ± 0.025	Increases from 24 h and remains stable at 4 weeks, returning to baseline at 12 weeks.	Increases from 24 h and remains stable at 4 weeks, returning to baseline at 12 weeks.
OPN	30.73 ± 72.82	No significant changes.	Increases with peak at 3 weeks.
PGE2	4.6 ± 0.8	Increases at 24 h and begins to decrease at 7 days.	Increases slightly at 24 h and begins to decrease at 7 days.
RANK	8.62 ± 15.27	Increases at 24 h, decreasing at 7 days.	Increases at 24 h, decreasing at 7 days.
CT	25.9 ± 25.9	Increases between 1 h and 7 days, remaining stable at 15 days.	No significant changes.
MMP-1	202.08 ± 132.63	Increases at 7 days.	Decreases at 7 days.
MMP-9	23,400.22 ± 22,986.03	Increase from 4 h to 7 days.	Increase from 4 h to 7 days.
SP	2.2 ± 0.9	Increases significantly at 24 h, decreases at 7 days.	Slight increase at 24 h and decrease at 7 days.

**Table 5:** Presence of biochemical markers in the different phases of bone regulation.

Phase	Bone Formation	Bone Resorption
**Early**	IL-27	NR
**Acute**	OPG TFG-β1 IL-10 OCN COL-1	RANKL TNF-α IL-1β IL-6 IL-17A IL-17F IL-23 PGE2 RANK SP
**Late**	NR	IL-17A IL-17F IL-23 OPN CT MMP-1 MMP-9

NR = Not Reported.

## Data Availability

No new data were created or analysed in this study. Data sharing is not applicable to this article.

## Supplementary Materials

The following supporting information can be downloaded at: https://www.mdpi.com/article/10.3390/jfb17010007/s1, S1: PubMed search strategy.

## Abbreviations

The following abbreviations are used in this manuscript:

OB	Osteoblast
OC	Osteoclast
OPG	Osteoprotegerin
PTH	Parathyroid hormone
CT	Calcitonin
GCF	Gingival Crevicular Fluid
PDL	Periodontal ligament
TGF-β1	Transforming Growth Factor β 1
IL-27	Interleukin 27
RANKL	Receptor Activator of Nuclear Factor Kappa β Ligand
TNF-α	Tumour Necrosis Factor-α
IL-1β	Interleukin 1β
IL-6	Interleukin 6
OPN	Osteopontin
PGE2	Prostaglandin E2
RANK	Receptor Activator of Nuclear Factor Kappa β
IL-17A	Interleukin 17A
IL-17F	Interleukin 17F
IL-23	Interleukin 23
MMP-1	Matrix Metallopeptidase 1
MMP-9	Matrix Metallopeptidase 9
SP	Substance P
OCN	Osteocalcin
COL-1	Collagen Type 1
TIMP-1	Metallopeptidase Inhibitor 1
TIMP-2	Metallopeptidase Inhibitor 2
IL-10	Interleukin 10
ALP	Alkaline Phosphatase
TRAP	Tartrate-Resistant Acid Phosphatase
TRAP5b	Tartrate-Resistant Acid Phosphatase 5b
IL-1α	Interleukin 1α
IL-2	Interleukin 2
IL-8	Interleukin 8
IL-13	Interleukin 13
IL-16	Interleukin 16

## References

[1] Fernández-Tresguerres Hernández-Gil I., Alobera Gracia M.Á., Del Canto Pingarrón M., Blanco Jerez L. (2006). Bases fisiológicas de la regeneración ósea II: El Proceso remodelado. Med. Oral. Patol. Oral. Cir. Bucal..

[2] Neyro Bilbao J., Cano Sánchez A., Palacios Gil-Antuñano S. (2011). Regulación del metabolismo óseo a través del sistema RANK-RANKL-OPG. Rev. Osteoporos. Metab. Min..

[3] Kim J.M., Lin C., Stavre Z., Greenblatt M.B., Shim J.H. (2020). Osteoblast-Osteoclast Communication and Bone Homeostasis. Cells.

[4] Ross M., Pawlina W. (2010). Histología: Texto y Atlas Color con Biología Celular y Molecular.

[5] Kanzaki H., Chiba M., Takahashi I., Haruyama N., Nishimura M., Mitani H. (2004). Local OPG gene transfer to periodontal tissue inhibits orthodontic tooth movement. J. Dent. Res..

[6] Nishida D., Arai A., Zhao L., Yang M., Nakamichi Y. (2021). RANKL/OPG ratio regulates odontoclastogenesis in damaged dental pulp. Sci. Rep..

[7] Calvo-Gallego J., Manchado-Morales P., Pivonka P., Martínez-Reina J. (2023). Spatio-temporal simulations of bone remodelling using a bone cell population model based on cell availability. Front. Bioeng. Biotechnol..

[8] Siddiqui J., Partridge N. (2016). Physiological Bone Remodeling: Systemic Regulation and Growth Factor Involvement. Physiology.

[9] Robins S., Brady J., Robins S.P., Brady J.D. (2002). Collagen Cross-Linking and Metabolism. Principles of Bone Biology.

[10] Riancho J.A., Delgado-Calle J. (2011). Mecanismos de interacción osteoblasto-osteoclasto. Reum. Clin..

[11] Zainal Ariffin S., Yamamoto Z., Zainol Abidin I., Megat Abdul Wahab R., Ariffin Z. (2011). Cellular and molecular changes in orthodontic tooth movement. Sci. World J..

[12] Trueta J. (1963). The role of blood vessels in osteogenesis. J. Bone Jt. Surg. Br..

[13] Hadjidakis D.J., Androulakis I.I. (2006). Bone Remodeling. Ann. N. Y Acad. Sci..

[14] Pérez Idarraga A. (2021). Medición de RANKL y OPG en Fluido Crevicular Durante el Tratamiento de Ortodoncia Invisalign^®^, con y sin Fuerzas Intermitentes Mediante Acceledent^®^.

[15] Reyes García R., Rozas Moreno P., Muñoz-Torres M. (2008). Regulación del proceso de remodelado óseo. REEMO.

[16] Zaidi M., Moonga B., Abe E. (2002). Calcitonin and bone formation: A knockout full of surprises. J. Clin. Investig..

[17] Karsdal M., Henriksen K., Arnold M., Christiansen C. (2008). Calcitonin: A drug of the past or for the future? Physiologic inhibition of bone resorption while sustaining osteoclast numbers improves bone quality. BioDrugs.

[18] Kumar A., Saravanan K., Kohila K., Kumar S. (2015). Biomarkers in orthodontic tooth movement. J. Pharm. Bioallied Sci..

[19] Meikle M. (2006). The tissue, cellular, and molecular regulation of orthodontic tooth movement: 100 years after Carl Sandstedt. Eur. J. Orthod..

[20] Kingsley N. (1880). A treatise on oral deformities as a branch of mechanical surgery. Am. J. Dent. Sci..

[21] Sandstedt C. (1904). Einige Beiträge zur Theorie der Zahnregulierung. Nord. Tandläkare Tidskr..

[22] Sandstedt C. (1905). Einige Beiträge zur Theorie der Zahnregulierung. Nord. Tandläkare Tidskr..

[23] Oppenheim A. (1911). Tissue changes, particularly of the bone, incident to tooth movement. Am. J. Orthod..

[24] Schwarz A. (1932). Tissue changes incidental to orthodontic tooth movement. Int. J. Orthod. Oral. Surg. Radiogr..

[25] Fukada E., Yasuda I. (1957). On the Piezoelectric Effect of Bone. J. Phys. Soc. Jpn..

[26] Brännström (1984). Communication between the oral cavity and the dental pulp associated with restorative treatment. Oper. Dent..

[27] Barbieri Petrelli G. (2016). Cambios Bioquímicos del Metabolismo Óseo Durante el Movimiento Ortodóncico.

[28] National Institute of Environmental Health Sciences https://www.niehs.nih.gov/health/topics/science/biomarkers.

[29] Jaraback J., Fizzell J. (1972). Technique and Treatment with Light-Wire Edgewise Appliances.

[30] Page M., McKenzie J., Bossuyt P., Boutron I., Hoffmann T., Mulrow C., Shamseer L., Tetzlaff J.M., Akl E.A., Brennan S.E. (2021). The PRISMA 2020 statement: An updated guideline for reporting systematic reviews. BMJ.

[31] Slim K., Nini E., Forestier D., Kwiatkowski F., Panis Y., Chipponi J. (2003). Methodological index for non-randomized studies (minors): Development and validation of a new instrument. ANZ J. Surg..

[32] Afacan B., Öztürk V.Ö., Geçgelen Cesur M., Köse T., Bostanci N. (2019). Effect of orthodontic force magnitude on cytokine networks in gingival crevicular fluid: A longitudinal randomized split-mouth study. Eur. J. Orthod..

[33] Alarcón J.A., Linde D., Barbieri G., Solano P., Caba O., Rios-Lugo M.J., Sanz M., Martin C. (2013). Calcitonin gingival crevicular fluid levels and pain discomfort during early orthodontic tooth movement in young patients. Arch. Oral. Biol..

[34] Alikhani M., Raptis M., Zoldan B., Sangsuwon C., Lee Y.B., Alyami B., Corpodian C., Barrera L.M., Alansari S., Khoo E. (2013). Effect of micro-osteoperforations on the rate of tooth movement. Am. J. Orthod. Dentofac. Orthop..

[35] Barbieri G., Solano P., Alarcón J.A., Vernal R., Rios-Lugo J., Sanz M., Martín C. (2013). Biochemical markers of bone metabolism in gingival crevicular fluid during early orthodontic tooth movement. Angle Orthod..

[36] Castroflorio T., Gamerro E.F., Caviglia G., Deregibus A. (2017). Biochemical markers of bone metabolism during early orthodontic tooth movement with aligners. Angle Orthod..

[37] Dudic A., Kiliaridis S., Mombelli A., Giannopoulou C. (2006). Composition changes in gingival crevicular fluid during orthodontic tooth movement: Comparisons between tension and compression sides. Eur. J. Oral. Sci..

[38] Garlet T.P., Coelho U., Silva J.S., Garlet G.P. (2007). Cytokine expression pattern in compression and tension sides of the periodontal ligament during orthodontic tooth movement in humans. Eur. J. Oral. Sci..

[39] Grant M., Wilson J., Rock P., Chapple I. (2013). Induction of cytokines, MMP9, TIMPs, RANKL and OPG during orthodontic tooth movement. Eur. J. Orthod..

[40] Karaduman B., Uraz A., Altan G., Tuncer B.B., Alkan Ö., Gönen S., Pehlivan S., Çetiner D. (2015). Changes of tumor necrosis factor-α, interleukin-10, and tartrate-resistant acid phosphatase5b in the crevicular fluid in relation to orthodontic movement. Eur. J. Inflamm..

[41] Kawasaki K., Takahashi T., Yamaguchi M., Kasai K. (2006). Effects of aging on RANKL and OPG levels in gingival crevicular fluid during orthodontic tooth movement. Orthod. Craniofacial Res..

[42] Lin T., Yang L., Zheng W., Zhang B. (2020). The Changes of Th17 Cytokines Expression and Its Correlation with Receptor Activator of Nuclear Factor Kappa B Ligand During Orthodontic Tooth Movement. Ir. J. Inmunol..

[43] Lee K.J., Park Y.C., Yu H.S., Choi S.H., Yoo Y.J. (2004). Effects of continuous and interrupted orthodontic force on interleukin-1beta and prostaglandin E2 production in gingival crevicular fluid. Am. J. Orthod. Dentofac. Orthop..

[44] Luppanapornlarp S., Kajii T.S., Surarit R., Iida J. (2010). Interleukin-1b levels, pain intensity, and tooth movement using two different magnitudes of continuous orthodontic force. Eur. J. Orthod..

[45] Otero L., García Dabeiba A., Wilches-Buitrago L. (2016). Expression and Presence of OPG and RANKL mRNA and Protein in Human Periodontal Ligament with Orthodontic Force. Gene Regul. Syst. Biol..

[46] Padisar P., Hashemi R., Naseh M., Nikfarjam B., Mohammadi M. (2018). Assessment of tumor necrosis factor alpha (TNFα) and interleukin 6 level in gingival crevicular fluid during orthodontic tooth movement: A randomized split-mouth clinical trial. Electron. Physician.

[47] Toygar H.U., Kircelli B.H., Bulut S., Sezgin N., Tasdelen B. (2008). Osteoprotegerin in gingival crevicular fluid under long-term continuous orthodontic force application. Angle Orthod..

[48] Tuncer B.B., Özdemir B.Ç., Boynueğri D., Karakaya I.B., Ergüder I., Yücel A.A., Aral L.A., Özmeriç N. (2013). OPG-RANKL levels after continuous orthodontic force. Gazi Med. J..

[49] Atkinson A., Colburn W., DeGruttola V., DeMets D., Downing G., Hoth D., Oates J.A., Peck C.C., Schooley R., Spilker B. (2001). Biomarkers and surrogate endpoints: Preferred definitions and conceptual framework. Clin. Pharmacol. Ther..

[50] Sobral De Aguilar M., Perinetti G., Capelli J.J. (2017). The Gingival Crevicular Fluid as a Source of Biomarkers to Enhance Efficiency of Orthodontic and Functional Treatment of Growing Patients. Biomed. Res. Int..

[51] Zavala-Jonguitud L.F., Anda J.C., Flores-Padilla M.G., Pérez C., Juárez-Villa J.D. (2021). Correlación entre la insuficiencia o deficiencia de los niveles de vitamina D y las interleucinas 1β y 6. Rev. Alerg. Mex..

[52] Franco L., Ortiz M. (2010). Marcadores bioquímicos del metabolismo óseo. Rev. Estomat..

[53] Vargas del Valle P., Piñeiro Becerra M., Palomino Montenegro H., Torres-Quintana M. (2010). Factores modificantes del movimiento dentario ortodóncico. Av. Odontoestomatol..

[54] Flórez-Moreno G.A., Isaza-Guzmán D.M., Tobón-Arroyave S.I. (2013). Time-related changes in salivary levels of the osteotropic factors sRANKL and OPG through orthodontic tooth movement. Am. J. Orthod. Dentofac. Orthop..

[55] Martiarena B., Kogovsek N., Salerni H., Castillo V., Brandi G., Regonat M., Visintini A., Quintana H., Otero P. (2015). Reference values of parathyroid hormone and vitamin D Hormone by chemiluminescent automated assay. RevMVZ Córdoba.

[56] Rody W.J., Wijegunasinghe M., Wiltshire W.A., Dufault B. (2014). Differences in the gingival crevicular fluid composition between adults and adolescents undergoing orthodontic treatment. Angle Orthod..

[57] Zhang Y., Zhou C., Li J., Zhang Y., Xie D., Liang M., Wang B., Song Y., Wang X., Huo Y. (2020). Serum alkaline phosphatase levels and the risk of new-onset diabetes in hypertensive adults. Cardiovasc. Diabetol..

[58] Sells Galvin R., Gatlin C., Horn J., Fuson T. (1999). TGF-beta enhances osteoclast differentiation in hematopoietic cell cultures stimulated with RANKL and M-CSF. Biochem. Biophys. Res. Commun..

[59] Wahab R.M.A., Abu Kasim N., Senafi S., Jemain A.A., Abidin I.Z.Z., Shahidan M., Ariffin S.H.Z. (2014). Enzyme Activity Profiles and ELISA Analysis of Biomarkers from Human Saliva and Gingival Crevicular Fluid during Orthodontic Tooth Movement Using Self-Ligating Brackets. Oral. Health Dent. Manag..

[60] Ono T., Hayashi M., Sasaki F., Nakashima T. (2020). RANKL biology: Bone metabolism, the immune system, and beyond. Inflamm. Regen..

[61] Nieto-Flores J., Villafán-Bernal J.R., Rivera-León E.A., Llamas-Covarrubias I.M., González-Hita M.E., Alcalá-Zermeno J.L., Alcalá-Zermeno J.L., Sánchez-Enríquez S. (2018). Niveles de referencia de osteocalcina en población sana de México. Gac. Med. Mex..

[62] Ren Y., Hazemeijer H., de Haan B., Qu N., de Vos P. (2007). Cytokine Profiles in Crevicular Fluid During Orthodontic Tooth Movement of Short and Long Durations. J. Periodontol..

[63] Atuğ Özcan S., Ceylan I., Ozcan E., Kurt N., Dağsuyu I., Canakçi C. (2014). Evaluation of Oxidative Stress Biomarkers in Patients with Fixed Orthodontic Appliances. Dis. Markers..

[64] Patil S., Gujar A., Baeshen H., Alhazmi A., Ghoussoub M.S., Raj A., Bhandi S., Sarode S., Awan K., Birkhed D. (2020). Comparison of Biochemical Markers of Bone Metabolism Between Conventional Labial and Lingual Fixed Orthodontic Appliances. Niger. J. Clin. Pr..

[65] Gameiro G.H., Schultz C., Trein M.P., Mundstock K.S., Weidlich P., Goularte J.F. (2015). Association among pain, masticatory performance, and proinflammatory cytokines in crevicular fluid during orthodontic treatment. Am. J. Orthod. Dentofac. Orthop..

[66] MacLaine J.K., Rabie A.B.M., Wong R. (2010). Does orthodontic tooth movement cause an elevation in systemic inflammatory markers?. Eur. J. Orthod..

[67] Reiss S., Chouinard M.C., Landa D.F., Nanda R., Chandhoke T., Sobue T., Allareddy V., Kuo C.-L., Mu J., Uribe F. (2020). Biomarkers of orthodontic tooth movement with fixed appliances and vibration appliance therapy: A pilot study. Eur. J. Orthod..

