# Supplementary material for: Biochemical Markers Involved in Bone Remodelling During Orthodontic Tooth Movement
Source: J Funct Biomater. 2025 Dec 22;17(1):7. doi: 10.3390/jfb17010007 (PMC12841841; doi:10.3390/jfb17010007)
Supplement: Supplementary file 1 [file jfb-17-00007-s001.zip › jfb-4019961-supplementary.pdf]

Journal of
Functional Biomaterials

Review

Biochemical Markers Involved in Bone Remodelling During Orthodontic Tooth Movement

Beatriz Patricia Fuentes Vera [1,*], Ibrahim Dib Zaitun [2] and María Ángeles Pérez de la Cruz [1]

[1] Department of Anatomy and Histology, University of Salamanca, 37003 Salamanca, Spain; mapec@usal.es
[2] Department of Surgery, University of Salamanca, 37003 Salamanca, Spain; ibrahimdib@usal.es
* Correspondence: dra.beatrizfuentes@gmail.com

Supplementary Material S1. PubMed search strategy.

Search: **(Biomarkers OR Markers OR Mediators) AND ("Orthodontic" OR "Dental Movement") NOT "Case Report"** Filters: **Humans**

((("biomarker s"[All Fields] OR "biomarkers"[Supplementary Concept] OR "biomarkers"[All Fields] OR "biomarker"[All Fields] OR "biomarkers"[MeSH Terms] OR ("marker"[All Fields] OR "markers"[All Fields]) OR ("mediated"[All Fields] OR "mediational"[All Fields] OR "mediator"[All Fields] OR "mediator s"[All Fields] OR "mediators"[All Fields] OR "negotiating"[MeSH Terms] OR "negotiating"[All Fields] OR "mediate"[All Fields] OR "mediates"[All Fields] OR "mediating"[All Fields] OR "mediation"[All Fields] OR "mediations"[All Fields])) AND ("Orthodontic"[All Fields] OR "Dental Movement"[All Fields])) NOT "Case Report"[All Fields]) AND (humans[Filter])

Translations

Biomarkers: "biomarker's"[All Fields] OR "biomarkers"[Supplementary Concept] OR "biomarkers"[All Fields] OR "biomarker"[All Fields] OR "biomarkers"[MeSH Terms]

Markers: "marker"[All Fields] OR "markers"[All Fields]

Mediators: "mediated"[All Fields] OR "mediational"[All Fields] OR "mediator"[All Fields] OR "mediator's"[All Fields] OR "mediators"[All Fields] OR "negotiating"[MeSH Terms] OR "negotiating"[All Fields] OR "mediate"[All Fields] OR "mediates"[All Fields] OR "mediating"[All Fields] OR "mediation"[All Fields] OR "mediations"[All Fields]
